# Learning Temporal Patterns of Risk in a Predator-Diverse Environment

**DOI:** 10.1371/journal.pone.0034535

**Published:** 2012-04-06

**Authors:** Yoland J. Bosiger, Oona M. Lonnstedt, Mark I. McCormick, Maud C. O. Ferrari

**Affiliations:** 1 ARC Centre of Excellence for Coral Reef Studies, and School of Marine and Tropical Biology, James Cook University, Townsville, Queensland, Australia; 2 Department of Biomedical Sciences, WCVM, University of Saskatchewan, Saskatoon, Saskatoon, Canada; Institute of Marine Research, Norway

## Abstract

Predation plays a major role in shaping prey behaviour. Temporal patterns of predation risk have been shown to drive daily activity and foraging patterns in prey. Yet the ability to respond to temporal patterns of predation risk in environments inhabited by highly diverse predator communities, such as rainforests and coral reefs, has received surprisingly little attention. In this study, we investigated whether juvenile marine fish, *Pomacentrus moluccensis* (lemon damselfish), have the ability to learn to adjust the intensity of their antipredator response to match the daily temporal patterns of predation risk they experience. Groups of lemon damselfish were exposed to one of two predictable temporal risk patterns for six days. “Morning risk” treatment prey were exposed to the odour of *Cephalopholis cyanostigma* (rockcod) paired with conspecific chemical alarm cues (simulating a rockcod present and feeding) during the morning, and rockcod odour only in the evening (simulating a rockcod present but not feeding). “Evening risk” treatment prey had the two stimuli presented to them in the opposite order. When tested individually for their response to rockcod odour alone, lemon damselfish from the morning risk treatment responded with a greater antipredator response intensity in the morning than in the evening. In contrast, those lemon damselfish previously exposed to the evening risk treatment subsequently responded with a greater antipredator response when tested in the evening. The results of this experiment demonstrate that *P. moluccensis* have the ability to learn temporal patterns of predation risk and can adjust their foraging patterns to match the threat posed by predators at a given time of day. Our results provide the first experimental demonstration of a mechanism by which prey in a complex, multi-predator environment can learn and respond to daily patterns of predation risk.

## Introduction

Predation shapes the behaviour, life history, morphology and distribution of prey animals over both evolutionary and ecological timescales [1], [2]. In order to survive, prey must carefully balance the costs of predator avoidance with the benefits of other fitness-promoting activities such as foraging and reproducing [3], [4]. To complicate matters, predators are highly variable in the threat they pose. Predator activity and hence risk to prey may vary depending on a predator's body size, their foraging preferences, and the place and time during which they focus their foraging effort [5].

On a temporal scale, the risk of being eaten may fluctuate on a seasonal, lunar, daily, or even a minute-by-minute basis [1], [6]. Yet there is some evidence that predation may be predictable enough to allow prey to adaptively respond to temporal patterns of predation risk [7]. For example, rodents [8], [9] and storm petrels [10] are known to reduce their activity during periods of bright moonlight when nocturnal predators are increasingly active. Ants, *Pheidole titanus*, avoid aboveground foraging activity during times of the day when their predators, the dipteran parasitoids are more active [11]. Also, predation pressure during dawn and dusk ‘crepuscular’ periods is thought to drive species specific sheltering times for diurnal reef fishes [12], [13] as well as the timing of group migration between resting and feeding areas in nocturnally foraging grunts (Haemulidae) and other fish species [14], [15]. Yet it is unknown whether these adjustments are a result of innate recognition or temporal threat-sensitive learning in ecological time [16], [17].

Many prey do not show innate recognition of their predators [18]. Learning provides a means by which prey can identify novel predators [19] and respond to changes in the predator community structure as it fluctuates through space and time [20]. Aquatic prey are known to rely on a number of sensory modalities to detect predation-related cues, and amongst them, chemosensory detection appears to be the most widespread [18]. More specifically, a wide diversity of aquatic taxa from corals to larval amphibians, rely on damage released chemical alarm cues (hereafter alarm cues) to assess the level of predation risk in their local environment [18]. Alarm cues are released from the damaged epidermis of prey animals attacked/captured by predators, hence providing a reliable indicator of predation threat to conspecifics and some heterospecifics [21]. Alarm cues can also mediate associative learning of predators via the simultaneous pairing with a predator cue (sight, smell or sound) [22]. Learned predator recognition mediated via alarm cues occurs in a wide variety of prey taxa [18].

The threat-sensitive predator avoidance hypothesis [5] predicts that prey should match the intensity of their antipredator response to the level of predation risk they experience. Recent studies demonstrate that learned predator recognition allows prey to gauge the level of threat posed by novel predators. For instance, predator-experienced fathead minnows have been shown to distinguish the diet [23], size [24], density and proximity [25] of predators using predator odour only. The first step for such threat-sensitive assessment is for prey to use the concentration of alarm cues as a proxy for risk assessment [18]. So far, threat sensitive learning in relation to temporal patterns of risk has been demonstrated in the embryos [26], [27] and larvae [16] of one amphibian, *Rana sylvatica*. Surprisingly little is known about the temporal foraging periodicity of predators and their prey in environments with highly complex predator assemblages, such as tropical rainforests and coral reefs.

Piscivorous fishes on coral reefs are abundant, diverse [28], and often distributed unevenly among habitat patches. [29]. Most reef fishes have a bipartite life cycle that includes a planktonic larval stage [30] and as a result, larvae rarely settle to a reef with the same composition and density of predators as their natal reef. As juveniles grow, they will enter the size selection ranges of new predators and so will need to maintain their ability to learn and adapt to novel predation risks [31], [32]. Being able to rapidly recognise the daily temporal foraging patterns of different predators should allow prey to maximise trade-offs between predator avoidance and foraging, leading to higher fitness and survival [16].

There is evidence that predation pressure as a whole on coral reefs may be non-uniform over 24 h periods [33], [34], [35]. Some predators are thought to have relatively predictable activity patterns, such as highly diurnal wrasses (Labridae) and trevally (Carangidae) or nocturnally active snappers (Lutjanidae) and grunts (Haemulidae) [36]. Predators may also forage during specific period of the day, for example, the lionfish, *Pterois volitans* which displays greater activity during dawn and dusk [37]. Hence, many coral reef fishes would benefit from learning the temporal foraging patterns of the predators commonly found in their habitat patches.

The current study investigates whether juvenile lemon damselfish, *Pomacentrus moluccensis*, can learn temporal patterns of risk, and subsequently respond in a threat-sensitive manner to reduce their risk of predation. Coral reef fishes have been shown to use alarm cues to learn about multiple unknown predators as well as the level of predation threat posed through associative learning [38], [39]. In the present study, groups of juvenile *P. moluccensis* (“morning-risk” groups hereafter) were conditioned with a predictable pattern of risk consisting of alarm cues paired with predator odour in the morning (predator feeding and dangerous – high-risk) and predator odour paired with seawater in the evening (predator present but not feeding – low-risk). Other groups (“evening-risk” groups) received the opposite treatment, i.e. a low-risk in the morning followed by a high-risk in the evening. After the conditioning period, we tested individual *P. moluccensis* of both groups for their response to the predator cue alone (in both the morning and the evening) to determine whether individuals displayed different antipredator responses based on the schedule of risk they experienced during the previous 6 days.

## Materials and Methods

### Ethics Statement

This research was undertaken with approval of the James Cook University animal ethics committee (permit: A1593) and according to the University's animal ethics guidelines.

### Study species

Our test species, juvenile *P. moluccensis* (Family Pomacentridae) are found in association with coral reefs throughout the Indo-Pacific and feed primarily on algae and zooplankton. *P. moluccensis* are particularly abundant on reefs around our study area, Lizard Island, Northern Great Barrier Reef, Australia (14°40′S, 145°28′E) and are preyed upon by multiple predators, including the blue spotted rockcod, *Cephalopholis cyanostigma* (Serranidae) [40]. *C. cyanostigma* are sedentary, highly site attached piscivores that are common throughout the Indo-Pacific and Lizard Island [40]. *C. cyanostigma* reach a maximum size of 35 cm [36] and are found on shallow protected reefs in association with damselfish [41]. Preliminary field observations suggest that rockcods in the family *Cephalopholis* exhibit crepuscular patterns of foraging and activity (pers obs) [42]. Therefore, there is the potential that *P. moluccensis* may be exposed to a predictable pattern of threat from *C. cyanostigma* throughout the day.

### Collection and maintenance

All fish were collected at Lizard Island from February to April 2011. Juvenile *P. moluccensis* (Total Length (TL); 29.1±4.3 mm; mean ± SD) were collected on shallow reefs around Lizard Island on SCUBA using hand nets and a solution of the anesthetic clove oil mixed with alcohol and seawater. Captured fish were transported back to Lizard Island Research Station where they were held in 16-l flow-through seawater aquaria (39×29×15 cm) under a 12∶12 light∶dark photoperiod at ambient seawater temperatures (28°C). Fish were acclimated for a minimum of 48 h before being used in experimental trials and were fed newly hatched *Artemia* sp. three times per day.

*C. cyanostigma* were collected using baited hook and line on snorkel. Fish were transported back to Lizard Island Research Station where they were acclimated for a minimum of 24 h. This allowed any prey fish faecal matter to pass through their digestive system and prevented contamination of the final predator stimulus [40]. Individual *C. cyanostigma* were placed in 68-l flow-through plastic holding tanks (60×36×39 cm) and were fed thawed squid once daily.

### Experimental overview

Our experiment involved two phases: a conditioning phase and a testing phase. Our conditioning protocol consisted of conditioning groups of 6 *P. moluccensis* with a predictable pattern of predation risk for 6 consecutive days. A conditioning period of this length was considered appropriate based on similar studies [43] and represented an ecologically relevant timeframe for the fish to establish predictability of a predation regime.

The experiment followed a randomised block design whereby each group of 6 fish (conditioned together in single conditioning tank) represented a block. Groups of *P. moluccensis* were randomly allocated to one of two conditioning risk treatments: morning-risk or evening-risk treatment. Morning risk treatment consisted of exposing groups of *P. moluccensis* to alarm cues paired with *C. cyanostigma* odour (hereafter, predator odour), in the morning (0630–0900 h) and seawater paired with predator odour in the evening (1600–1830 h). The evening-risk treatment involved the opposite stimuli being given during morning and evening. The same times where used as for the morning-risk groups. As a result of constraints in time and tank availability during the testing stage, the total number of groups conditioned at one time needed to be staggered across different days. Every day, a new set of 4 groups (a total of 24 fish) would start their 6-day conditioning period. Of these 4 groups, 2 groups received the morning-risk treatment and 2 groups received the evening-risk treatment.

Following the 6-day conditioning period, 24 *P. moluccensis* (two morning risk groups and two evening risk groups) were transferred individually into observation tanks for testing. After a 24 h acclimation period, *P. moluccensis* were tested either in the morning (morning testing treatment: 0630–0900 h) or in the evening (evening testing treatment: 1600–1830 h) for their response to one of three testing stimuli: alarm cues, predator odour, or seawater. Conspecific alarm cues were used as a positive control, given that fish should always respond to risk cues regardless of time [18]. Seawater was used as a negative control to account for any disturbance as a result of introducing stimulus into testing tanks.

We tested approximately 18 individual *P. moluccensis* for each of our 12 treatments (2 conditioning risk treatments, 2 testing times, and 3 testing stimuli). All conditioning and testing protocols were conducted outside to ensure that prey fish could access all potentially necessary temporal cues (sun position, temperature).

### Stimulus preparation

Fresh alarm cues were prepared daily prior to the conditioning phase using juvenile *P. moluccensis* (TL; 29.1±4.3 mm; mean ± SD). To ensure that alarm cues were of sufficient potency, 3 *P. moluccensis* were used to make alarm cues for two subsequent trials. Individual *P. moluccensis* were euthanised by cold shock and were subsequently placed into a clean Petri dish. A scalpel blade was used to make 12 superficial vertical incisions (minor flesh damage) along each flank of each donor fish. Groups of 3 *P. moluccensis* were rinsed together in 20 ml of seawater (6.67 ml per fish) to average any differences in body size. The solution was then gently mixed for 30 s using a vortexer and subsequently filtered through filter paper in order to remove any solid matter, which could potentially initiate a feeding response.

Predator odour was obtained from *C. cyanostigma* by leaving individual fish in separate 68-l flow-through plastic holding tanks (60×36×39 cm) filled with 30-l of aerated seawater. Two pairs of *C. cyanostigma* (TL; 270 and 250 mm, and 290 and 250 mm) were placed on staggered alternating cycles of 12 h water flow on and approximately 56 h water flow off, to ensure that predator odour was consistently available for experimental use, and stress to individual *C. cyanostigma* was reduced. Following the cessation of water flow for 56 h, predator odour was prepared by drawing up 20 ml of predator water into a syringe. 10 ml of predator water was drawn from each predator tank within a pair to avoid intraspecific predator variability effects. Fish were fed squid once daily and tanks were cleaned to remove any excess matter such as faeces on days when water flow was returned to aquaria.

### Observation tanks

Groups of *P. moluccensis* were conditioned in 6-l flow-through aquaria (24×16×17 cm). Each conditioning tank had a 2 cm layer of sand and 5 pieces of plastic tubing to reduce any aggressive interactions between *P. moluccensis*. After the final conditioning treatment, individual *P. moluccensis* were transferred into 14-l flow-through aquaria (38×24×27 cm) for testing. A single airstone was attached to the right side of both conditioning and testing tanks and was joined to two 1.5 m long plastic tubes: one for injecting food and one for injecting the stimulus. Plastic tube ends were attached approximately 1 cm above the airstone to allow for rapid dispersal of food and/or stimulus into the aquaria. Each testing tank had a 2 cm layer of sand and an artificial branching *Acropora* coral (moulded resin; item no. 21505; Wardleys/TFH; Sydney; dimensions: 14×11.5×5 cm) placed on the opposite side of the aquarium to the stimulus injection tube to create a vertical shelter. In both conditioning and testing tanks, three sides were wrapped in black plastic and tanks were positioned behind a plastic observation blind to minimise observer disturbance to fish.

Conditioning treatments (morning-risk or evening-risk) were systematically allocated among conditioning tanks to ensure that the position of tanks did not confound results. All treatments (conditioning risk, testing time, and testing stimulus) were randomly allocated among testing tanks.

### Conditioning procedure

Groups of 6 individual *P. moluccensis* were exposed to either morning-risk or evening-risk. The ‘alarm cues paired with predator odour’ stimuli consisted of injecting 10 ml of conspecific *P. moluccensis* alarm cues paired with 20 ml of *C.* predator odour in each tank. The ‘seawater paired with predator odour’ stimuli consisted of injecting 10 ml of seawater paired with 20 ml of predator odour into each tank.

During the conditioning phase, groups of *P. moluccensis* were fed *ad libitum* three times daily with newly hatched *Artemia* sp.. Food was never given less than 1 h from the most recent conditioning to ensure that fish did not associate injection of stimuli with receiving food. Prior to the injection of the stimuli (either alarm cues or seawater paired with predator odour), the water flow was stopped and 60 ml of water was drawn out and discarded to remove any stagnant water from within the stimulus injection tube. For the ‘seawater paired with predator odour’ stimuli, an additional 10 ml of seawater was drawn out into a syringe prior to the stimuli being injected to act as the seawater component. A final 60 ml of seawater was then drawn out and retained in order to flush the stimuli (either alarm cues or seawater paired with predator odour) into the conditioning tanks.

### Testing procedure

The behaviour of focal damselfish was quantified for 3 min before (pre-stimulus period) and 3 min after (post-stimulus period) the addition of the testing stimuli (alarm cues, predator odour or seawater). Approximately 2 min prior to the start of the trial, the water flow was stopped and 60 ml of water was drawn out and discarded to remove any stagnant water from within the stimulus injection tube. A further three 60 ml portions of seawater was drawn out and retained to ensure that food/stimulus could be flushed into the tank. If a seawater stimulus was being injected, an extra 60 ml portion was drawn out to act as the stimulus. Prior to the pre-stimulus period, 2.5 ml (approx. 160 nauplii per ml) of *Artemia* sp. was added to the aquaria to stimulate feeding. After food was injected, test fish were left to feed for 1 min to allow fish to reach a stable feeding rate. The behaviour of a single juvenile *P. moluccensis* was then recorded for 3 min. Immediately following the pre-stimulus period, another 2.5 ml of extra *Artemia* sp. was added to the testing tank to maintain constant feeding levels. After 1 min of feeding, 10 ml of experimental stimulus was injected into the tank and the behaviour of fish was recorded for a further 3 min (post-stimulus period). During observation periods, observers were blind to the conditioning treatment to which *P. moluccensis* had previously been exposed.

### Behavioural assay

During both the pre-stimulus and the post-stimulus observation periods, three different behavioural traits were recorded as indicators of antipredator response: foraging rate, activity level, and distance from shelter. Reduced foraging rate and activity level, and increased shelter use are all common antipredator responses in a variety of taxa including coral reef fishes [38], [39]. Foraging rate was recorded as the total number of strikes within the 3-min period, irrespective of whether fish were successful at capturing food items. To determine the activity and distance from shelter, a 4×4 cm grid was drawn onto tanks, making up a total of 32 individual squares. Each time an individual fish crossed one of the lines marked on the tank it was recorded, giving a measure of activity level. The vertical lines also represented distinct distances away from the coral shelter, with the shelter filling the first zone (laterally) and a fish thus considered 0 cm from shelter when within it. The second zone was 0–4 cm from shelter, and the remaining three zones 4–12, 12–20, and 20–28 cm from shelter. The total time spent within each of the 5 zones was estimated on conclusion of the 3-min observation. Mean maximum distance from shelter was calculated from the cumulative proportion of time spent in each zone.

### Statistical analysis

Statistical comparisons were conducted on the percent change in behavioural measure from the pre-stimulus baseline: (post-stimulus value - pre-stimulus value)/(pre-stimulus value). While conditioning group was originally introduced as a random factor, this factor was subsequently removed after preliminary statistical analyses revealed that it did not have any significant effect on the analysis [44].

The data (N = 210) were examined for outliers and one influential point was removed. Residual analysis suggested that data were normally distributed, however, the variances were not homogeneous among treatments. We therefore analysed the data using non-parametric MANOVA and ANOVAs on the rank value of the data [27], [44]. As the three response variables (foraging, line crosses and relative distance from shelter) were not independent from each other, a 3-factor non-parametric MANOVA was used to analyse the effect of conditioning risk (morning-risk or evening-risk), testing time (morning or evening) and testing stimulus (alarm cue, predator odour or seawater) on behaviour. This was followed by 3-factor non-parametric ANOVAs on each response variable separately.

Because of a significant 3-factor interactions between conditioning risk, testing time and testing stimulus, the response of fish to conditioning risk and testing time was then analysed using 2-factor non-parametric ANOVAs on each testing stimuli separately (alarm cue, predator odour and seawater), followed by Tukey's HSD post hoc comparisons.

## Results

The non-parametric 3-factor MANOVA revealed a statistically significant interaction among the conditioning treatment, testing time and testing stimulus (Pillai's trace:_6, 394_ = 0.08, p<0.05). The 3-factor ANOVAs revealed a significant 3-factor interaction between conditioning treatment, testing time and testing stimulus for each of the variables: foraging rate (F_2, 198_ = 5.03, p<0.01), line crosses (F_2, 198_ = 3.06, p<0.05) and relative distance from shelter (F_2, 198_ = 3.52, p<0.05).

To investigate the nature of the interaction, analyses were performed on each cue separately. No statistically significant interaction between conditioning risk and testing time was found for *P. moluccensis* tested with alarm cues or seawater for any of the response variables (p>0.05). In other words, fish constantly responded to the risk cues and never responded to the control cues, regardless of conditioning risk or testing time. In contrast, a statistically significant interaction was apparent between conditioning and testing time for prey fish tested with predator odour: foraging (F_1, 67_ = 20.42, p<0.001), line crosses (F_1, 67_ = 6.50, p<0.05), and relative distance (F_1, 67_ = 8.88, p<0.005).

**Figure 1:**
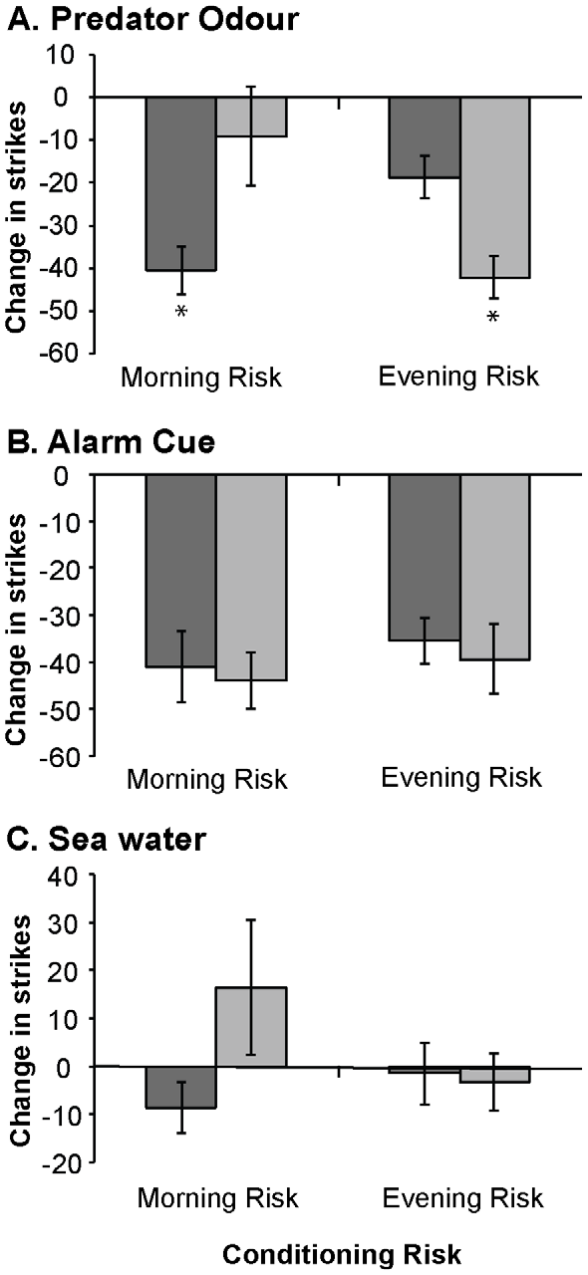
Change in foraging rate for *Pomacentrus moluccensis* in response to cues of varying threat. Mean (± SE) percentage change in strike rate (per 3-min observation) from the pre-stimulus baseline for *Pomacentrus moluccensis* tested with: A. predator odour (PO), B. alarm cues (AC) or C. seawater (SW) at one of two times: morning (dark grey bars) or evening (light grey bars). *P.moluccensis* were previously conditioned for six days with either morning risk (high-risk in the morning, low-risk in the evening) or evening risk (high-risk in the evening, low-risk in the morning). * Indicate significant differences at p<0.05, between *P. moluccensis* tested in the morning and evening.

Tukey's HSD post-hoc comparisons revealed that fish previously exposed to both morning and evening conditioning treatments exhibited a statistically significant (p<0.05) decrease in foraging at the testing time in which they had previously been conditioned with risk, as opposed to the testing time in which they had previously been conditioned with predator odour alone (Fig. 1). The following discussion thus focuses on foraging rate, as this was the only variable for which a statistically supported description of the response pattern was possible.

## Discussion

The present study shows that juvenile coral reef fish have the ability to learn to respond to temporal patterns of predation risk. This experiment clearly establishes that *P. moluccensis* have the capacity to develop threat-sensitive responses to predator contingent on the temporal pattern of predation risk that they receive. Individual *P. moluccensis* showed a significant reduction in foraging to predator odour when tested at the time of day to which they had previously been conditioned to receive higher risk. In contrast, those *P. moluccensis* tested with alarm cues alone did not vary their foraging response in respect to conditioning and testing treatments, highlighting the importance of alarm cues as perpetual indicators of imminent risk [18].

*P. moluccensis* did not display a significant reduction in line crosses or distance from shelter when presented with predator odour at the time of risk conditioning, although the pattern of reduction in these measures was similar to that found for foraging rate. Therefore, it appears that in contrast to foraging, reduced activity level and distance from shelter were not key antipredator responses for juvenile *P. moluccensis* in this instance.

Learning temporal patterns of risk has been reported in amphibians [16], [17], however this is the first study to identify the associative learning of temporal patterns in a marine fish. This is also the first study to investigate temporal associative learning in a highly predator diverse environment.

Like most prey species, coral reef fish must effectively balance predator avoidance with other fitness-promoting behaviours (i.e. foraging) [1]. The threat-sensitive predator avoidance hypothesis suggests that prey should trade-off predator avoidance against other fitness promoting activities, and should do so in an appropriate graded manner [5].

The results of the current experiment uphold predictions from the threat sensitive avoidance hypothesis as prey demonstrated a stronger antipredator response to the introduction of predator odour when the time of testing corresponded with the time of high-risk conditioning. The response was categorised by a substantial decrease in foraging rate, a common antipredator behaviour exhibited by a number of species including *P. moluccensis* [39]. Given the cost involved in reducing foraging effort, the behaviour displayed by *P. moluccensis* suggests that being vigilant during a predator's preferred foraging time may be a small price to pay in comparison to the probable benefit of reducing mortality [26].

Like most demersal marine fishes, *P. moluccensis* has a dispersive larval stage in the pelagic environment, and settle on the reef around the time of metamorphosis [30]. Learning of resident predator species is likely to be critical at this life history transition where mortality is extremely high [45], [46]. However, juvenile *P. moluccensis* in our study were already established on the reef for ∼1 month and are therefore likely to have had prior knowledge of temporal foraging and activity patterns exhibited by *C. cyansostigma*. To account for this, prey fish were randomly assigned to one of two conditioning risk treatments, which provided high-risk either in the morning or evening. This design allowed us to conclude with confidence that the behaviour of juvenile *P. moluccensis* was a result of learning at the time of experimentation and not due to innate or previously learned temporal behaviour of predators.

Temporal threat sensitive learning has been explored in one other species of fish: *Galaxias muculatus* [47]. Reebs (1999) found that *G. muculatus* were unable to learn to be in a specific time and place to escape predation (time-place learning), raising questions about whether predators are predictable enough both in space and time to allow learning to occur [17], [48]. In contrast, the current study shows that learning temporal patterns of risk can develop in juvenile *P. moluccensis* within a period of six days. While little was known about the nature of predation risk in the study by Reebs (1999), common resident predators of *P. moluccensis*, such as *P. fuscus* and *Cephalopholis* spp. do have foraging peaks during particular times of the day (Feeney et al. submitted; Fishelson et al. 1989). Both *P. fuscus* and *C. cyanostigma* are sedentary, patchily distributed, and are known to occupy relatively small territories in the vicinity of planktivorous damselfish [29], [41]. Similarly, juvenile *P. moluccensis* are extremely site attached with tagging studies showing that fish rarely move further than 2 m [49]. While more transient fishes may be inclined to shift their foraging habitat to avoid predators, juvenile *P. moluccensis* will be forced to live with any predator species that takes up residence in their habitat patch. This situation is likely to promote shifts in foraging time, which oppose the diel foraging pattern of predators.

Learning is thought to be advantageous for prey where the environment may fluctuate and predator composition vary [48], [50]. In a complex environment such as a coral reef, it is highly likely that predation risk may change through time [1], [6]. One way that coral reef fishes will experience variation in predation threat is through immigration and emigration of resident predators from their habitat patch. For instance, even *C. cyanostigma*, a relatively site attached predator, may move distances of between 21 and 48 m over longer time scales (three months to two years) [41]. As diel foraging patterns vary among predator species, a flexible mechanism allowing prey to respond to temporal variability in predation risk would be highly beneficial.

In complex coral reef environments, it is also possible that predators may temporarily alter their predominant foraging patterns even whilst remaining within their habitat patches. If we consider that both predators and prey are active participants in behavioural interactions there may come a point where too much predictability will be detrimental for predators that specialise in few prey species [51]. Notwithstanding any physiological constraints, predators may increase variation in their temporal foraging patterns to increase success. For example, recent studies by Roth and Lima [51] have found that hawks need to attack at random if they are ever to successfully catch pigeons. Predator-prey interactions can thus be likened to a dynamic behavioural game, whereby if risk is too predictable, prey will be able to learn too efficiently, eventually causing predators to change their attack schedule to be more efficient [51], [52].

There may also be particular times of the day that are simply more dangerous for diurnal reef fishes as a whole. On coral reefs, predation pressure is considered to be strongest during dawn and dusk [33], [34], [53] with predators thought to possess physiological adaptations for operating at lower light levels [54]. Diurnal reef fishes, such as juvenile *P. moluccensis*, may therefore find it innately easier to respond to predation risk provided at dawn and dusk than other times within the diel cycle. We strongly encourage further studies that investigate species-specific diel foraging patterns within coral reefs and other complex systems to allow us to determine any overarching patterns.

While various studies have investigated the effect of temporal patterns in predation risk on prey, a largely unappreciated aspect of the relationship concerns how prey allocate behaviours when temporal risk varies [6]. The risk allocation hypothesis (RAH) is one of the first models to actually consider how temporal variation in predation risk through time (days, weeks or months) may influence prey antipredator behaviour [6]. In contrast to the threat sensitive model, the RAH looks specifically at how background levels of predation risk over days to weeks may influence how prey allocate their foraging efforts and hence exposure to predation risk [6]. While we did not specifically test the RAH in this study, the ability for prey animals to assess temporal predation risk through learning is likely crucial for them to display effective and cost-efficient antipredator responses, including strategies following the RAH. Further studies on how coral reef fishes allocate their risk-taking behaviours through time will be essential if we are to achieve a holistic understanding of temporal periodicity in risk and its effect on predator-prey dynamics in complex ecosystems.

Despite the importance of diel patterns of predation risk in influencing prey behaviour, the link between predator and prey activity schedules has often only been inferred, and in very few cases has the mechanism maintaining this link been determined [7], [16]. Our study provides some of the first evidence that even in a highly complex system, prey have the ability to learn and aptly respond to predictable temporal patterns of predation risk. Although we have only begun to scratch the surface of how pervasive and important these effects may be, such abilities may play an important role in structuring prey behaviour in a wide range of ecosystems.

## References

[1] Lima SL, Dill LM (1990). Behavioral decisions made under the risk of predation: a review and prospectus.. Can J Zool.

[2] Smith RJF, Godin JJ (1997). Avoiding and deterring predators. Behavioural Ecology of Teleost Fishes.

[3] Milinski M, Pitcher TJ (1993). Predation risk and feeding behaviour.. Behaviour of teleost fishes.

[4] Sih A (1980). Optimal behaviour: can foragers balance two conflicting demands?. Science.

[5] Helfman GS (1989). Threat-sensitive predator avoidance in damselfish-trumpetfish interactions.. Behav Ecol Sociobiol.

[6] Lima SL, Bednekoff PA (1999). Temporal variation in danger drives antipredator behavior: the predation risk allocation hypothesis.. Am Nat.

[7] Kronfeld-Schor N, Dayan T (2003). Partitioning of time as an ecological resource.. Annu Rev Ecol Syst.

[8] Kotler BP, Brown JS, Dall SRX, Gresser S, Ganey D (2002). Foraging games between gerbils and their predators: temporal dynamics of resource depletion and apprehension in gerbils.. Evol Ecol Res.

[9] Clarke JA (1983). Moonlight's influence on predator/prey interactions between short-eared owls (*Asio flammeus*) and deermice (*Peromyscus maniculatus*).. Behav Ecol Sociobiol.

[10] Watanuki Y (1986). Moonlight avoidance behavior in Leach's storm-petrels as a defense against slaty-backed gulls.. The Auk.

[11] Feener DH (1988). Effects of parasites on foraging and defense behavior of a termitophagous ant, *Pheidole titanis* Wheeler (Hymenoptera:Formicidae).. Behav Ecol Sociobiol.

[12] Domm SB, Domm AJ (1973). The sequence of appearance at dawn and disappearance at dusk of some coral reef fishes.. Pac Sci.

[13] Helfman GS (1978). Patterns of community structure in fishes: summary and overview.. Environ Biol Fishes.

[14] Galdfelter WB (1979). Twilight migrations and foraging activities of the copper sweeper *Pempheris schomburgki* (Teleostei: Pempheridae).. Mar Biol.

[15] Helfman GS, Meyer JL, McFarland WN (1982). The ontogeny of twilight migration patterns in grunts (Pisces: Haemulidae).. Anim Behav.

[16] Ferrari MCO, Messier F, Chivers DP (2008). Larval amphibians learn to match antipredator response intensity to temporal patterns of risk.. Behav Ecol.

[17] Ferrari MCO, Chivers DP (2009). Temporal variability, threat sensitivity and conflicting information about the nature of risk: understanding the dynamics of tadpole antipredator behaviour.. Anim Behav.

[18] Ferrari MCO, Wisenden BD, Chivers DP (2010). Chemical ecology of predator-prey interactions in aquatic ecosystems: a review and prospectus.. Can J Zool.

[19] Brown GE (2003). Learning about danger: chemical alarm cues and local risk assessment in prey fishes.. Fish Fish.

[20] Kelly JL, Magurran AE (2003). Learned predator recognition and antipredator responses in fishes.. Fish Fish.

[21] Chivers DP, Smith RJF (1998). Chemical alarm signalling in aquatic predator-prey systems: a review and prospectus.. Ecoscience.

[22] Suboski MD (1990). Releaser-induced recognition learning.. Psychological Review.

[23] Mathis A, Smith RJF (1993). Fathead minnows, *Pimephales promelas*, learn to recognize northern pike, *Esox lucius*, as predators on the basis of chemical stimuli from minnows in the pike's diet.. Anim Behav.

[24] Kusch RC, Mirza RS, Chivers DP (2004). Making sense of predator scents: investigating the sophistication of predator assessment abilities of fathead minnows.. Behav Ecol Sociobiol.

[25] Ferrari MCO, Messier F, Chivers DP (2006). The nose knows: minnows determine predator proximity and density through detection of predator odours.. Anim Behav.

[26] Ferrari MCO, Manek AK, Chivers DP (2010). Temporal learning of predation risk by embryonic amphibians.. Biol Lett.

[27] Ferrari MCO, Chivers DP (2009). The ghost of predation future: threat-sensitive and temporal assessment of risk by embryonic woodfrogs.. Behav Ecol Sociobiol.

[28] Hixon MA, Sale PF (1991). Predation as a process structuring coral reef fish communities.. The Ecology of Fishes on Coral Reefs.

[29] Beukers JS, Jones GP (1997). Habitat complexity modifies the impact of piscivores on a coral reef fish population.. Oecologia.

[30] Leis JM, McCormick MI, Sale PF (2002). The biology, behaviour, and ecology of the pelagic, larval stage of coral reef fishes.. Coral Reef Fishes - Dynamics and Diversity in a Complex Ecosystem.

[31] Holmes TH, McCormick MI (2010). Size-selectivity of predatory reef fish on juvenile prey.. Mar Ecol Prog Ser.

[32] Lönnstedt OM, McCormick MI (2011). Chemical alarm cues inform prey of predation threat: the importance of ontogeny and concentration in a coral reef fish.. Anim Behav.

[33] Holbrook SJ, Schmitt RJ (2002). Competition for shelter space causes density-dependent predation mortality in damselfishes.. Ecology.

[34] Danilowicz BS, Sale PF (1999). Relative intensity of predation on the French grunt, *Haemulon flavolineatum*, during diurnal, dusk, and nocturnal periods on a coral reef.. Mar Biol.

[35] Helfman GS, Pitcher TJ (1993). Fish behaviour by day, night and twilight.. Behaviour of Teleost Fishes.

[36] Randall JE, Allen GR, Steene RC (1997). Fishes of the Great Barrer Reef and Coral Sea.

[37] Green SJ, Akins JL, Côte IM (2011). Foraging behaviour and prey consumption in the Indo-Pacific lionfish on Bahamian coral reefs.. Mar Ecol Prog Ser.

[38] Holmes TH, McCormick MI (2010). Smell, learn and live: The role of chemical alarm cues in predator learning during early life history in a marine fish.. Behav Processes.

[39] Mitchell MD, McCormick MI, Ferrari MCO, Chivers DP (2011). Coral reef fish rapidly learn to identify multiple unknown predators upon recruitment to the reef.. PloS One.

[40] Beukers-Stewart BD, Jones GP (2004). The influence of prey abundance on the feeding ecology of two piscivorous species of coral reef fish.. J Exp Mar Biol Ecol.

[41] Stewart BD, Jones GP (2001). Associations between the abundance of piscivorous fishes and their prey on coral reefs: implications for prey-fish mortality.. Mar Biol.

[42] Shpigel M, Fishelson L (1989). Food habits and prey selection of three species of groupers from the genus Cephalopholis (*Serranidae: Teleostei*).. Environ Biol Fishes.

[43] Ferrari MCO, Rive AC, MacNaughton CJ, Brown GE, Chivers DP (2008). Fixed vs. random temporal predictability of predation risk: an extension of the risk allocation hypothesis.. Ethology.

[44] Sokal RR, Rohlf FJ (2003). Biometry: the principles and practice of statistics in biological research.

[45] Almany G, Webster M (2006). The predation gauntlet: early post-settlement mortality in reef fishes.. Coral Reefs.

[46] Doherty PJ, Dufour V, Galzin R, Hixon MA, Meekan MG (2004). High mortality during settlement is a population bottleneck for a tropical surgeonfish.. Ecology.

[47] Reebs SG (1999). Time-place learning based on food but not predation risk in a fish, the Inanga (*Galaxias maculatus*).. Ethology.

[48] Stephens DW (1991). Change, regularity, and value in the evolution of animal learning.. Behav Ecol.

[49] Mapstone BD (1988). Patterns in the abundance of *Pomacentrus moluccensis* (Bleeker)..

[50] Slobodkin LB, Rapoport A (1974). An optimum strategy of evolution.. Q Rev Biol.

[51] Roth TC, Lima SL (2007). Use of prey hotspots by an avian predator: purposeful unpredictability?. Am Nat.

[52] Lima SL (2002). Putting predators back into behavioral predator-prey interactions.. Trends Ecol Evol.

[53] Hobson ES (1965). Diurnal-nocturnal activity of some inshore fishes in the gulf of California.. Copeia.

[54] Munz FW, McFarland WN (1973). The significance of spectral position in the rhodopsins of tropical marine fishes.. Vision Res.

